# Association of Dietary Patterns with Metabolic Syndrome

**DOI:** 10.3390/nu12092840

**Published:** 2020-09-17

**Authors:** Nicola Di Daniele

**Affiliations:** UOC of Internal Medicine-Center of Hypertension and Nephrology Unit, Department of Systems Medicine, University of Rome Tor Vergata, Via Montpellier 1, 00133 Rome, Italy; didaniele@med.uniroma2.it; Tel.: +39-062090-2982; Fax: +39-062090-3362

**Keywords:** metabolic syndrome, Mediterranean diet, dietary model, obesity

Metabolic syndrome (MetS), as originally defined by Jean Vague in 1940, was identified as a cluster of chronic, inflammatory pathologies, such as arterial hypertension, abdominal obesity, high glucose levels, high triglyceride levels and low HDL levels in the blood [1]. The definition of MetS as a combination of conditions that act together has subsequently been confirmed in the various classifications of the World Health Organization (WHO), the National Cholesterol Education Program Adult Treatment Panel III (NCEP ATPIII) and the International Diabetes Federation (IDF) [2]. It therefore naturally follows that the predisposition for the development of MetS, that by its very definition affects metabolic homeostasis, and which is unfortunately increasing in Western countries (WHO data indicate the prevalence of MetS in the adult population worldwide as being about 34%) [3], is strongly influenced by an imbalance between energy intake and energy expenditure. It therefore also follows that diet and the various “*dietary models*” play a fundamental role in the development of MetS. Scientific discoveries have amply demonstrated how a dietary regimen involving mere caloric restriction is alone able to not only prevent but also to favorably reverse the various pathological aspects of MetS [4]. The results from studies performed by Hammer and colleagues [5] report how substantial weight loss, due to significant caloric restriction in obese patients, was able to reduce both the levels of circulating glucose and saturated fatty acids in the blood, as well as the levels of circulating pro-inflammatory cytokines and leptin levels, demonstrating a basic association between caloric intake and the pathogenesis of MetS. The beneficial action of a hypocaloric diet on MetS is mainly due to a combined action at the multisystem level that simultaneously has an effect on insulin resistance, on pancreatic β-cell dysfunction, on the lipid profile and on organ damage mediated by the accumulation of visceral fat. However, MetS, by its very definition, is a clustering of at least three of the five possible medical conditions that are associated with the syndrome. As a consequence, the adoption of specific dietary patterns, that can reduce the risk of the various chronic diseases associated with MetS, can play an even more significant role than mere caloric restriction. For example, just as a high consumption of carbohydrates has been associated with an increased risk of MetS, due to its adverse effects on insulin resistance and β-cell dysfunction [6], it is also true that adherence to dietary patterns, such as the classic Mediterranean diet (MD), which is rich in fish, fruits, legumes, low-fat dairy products, extra virgin olive oil, red wine and fibre, has been shown to have a significantly beneficial effect on MetS and on the chronic diseases that characterize it [7]. Panagiotakos and colleagues in the ATTICA study analyzed the impact of the MD on the prevalence of MetS in 3042 men and women, demonstrating how this diet was able to significantly reduce the risk of MetS and the cardiovascular diseases associated with it [8]. The main beneficial effects of the MD for MetS have been shown to be associated with its high antioxidant and anti-inflammatory capacity. In fact, in all the pathologies associated with MetS, a low level of chronic inflammation, as well as the presence of oxidative stress (OS), play a predominant role in organ damage [9,10,11]. Omega-3, oleic acid, and polyphenols, such as resveratrol, contained in red wine grapes, are all compounds that act on different molecular mechanisms and processes that are capable of reducing the production of pro-inflammatory cytokines, such as TNF-α, ILβ and IL6 and the synthesis of reactive oxygen species (ROS) and to activate antioxidant pathways such as that of the seven sirtuins that are also able to control the cycle of hormones of insulin metabolism, such as IGF-1 [12]. In addition to these compounds, ascorbate, tyrosol, quercetin, catechin and tocopherol have been shown to be other bioactive nutrients that have a beneficial effect for MetS [12].

The MD and simple caloric restriction are not the only dietary models used for treating this inflammatory-metabolic disorder, even though, up to now, they have shown to be the most effective in terms of prevention against MetS. The CHO diet, based on the quality of carbohydrates as ranked on the glycaemic index (GI) and on their insulin response, has been recommended as a dietary strategy to control the negative prognostic factors of MetS [13]. Similarly, the moderate–high protein content diet has been recommended to regulate satiety, the energy expenditure processes, thermogenesis and gluconeogenesis, which are all mechanisms underlying the pathophysiology of MetS [14]. Another dietary pattern that has been proposed as a way of controlling the various MetS risk components involves an increase in meal frequency (high meal frequency pattern) for better caloric control that is associated with faster gastric emptying and controlled weight loss [15]. However, even if these dietary models have proven to be effective in the prevention and treatment of MetS, the ultimate goal is to find a dietary pattern, the most effective one possible, where functional nutrients are associated with a dietary strategy that is able to significantly reduce inflammation and OS, which are the underlying mechanisms of all the chronic disorders associated with MetS. An innovative strategy, in this regard, is to use a dietary model that is capable of modifying the intestinal microbiota, as the bacteria present in the intestine have been shown to influence the levels of important metabolites, such as adipokines and chemokines. These are then able to regulate the level of chronic inflammation and OS and consequently have an effect on the various risk factors that characterize MetS [16,17].

In conclusion, MetS is a complex and multi-factorial syndrome with a global incidence that is increasing, especially in Western countries. As a syndrome that is characterized by the presence of several chronic pathologies, this means that establishing a proper therapeutic strategy is complicated and in no way conclusive. It is therefore prevention that is of fundamental importance, and in particular diet and dietary models, meaning the potential benefits of the diet itself and the subsequent effects of the nutrients on the pathophysiology of the syndrome. Up to now, diet combined with adequate physical activity appears to be the only possible preventive therapy against MetS and, as with all therapies, the more personalized the diet, the more effective it will turn out to be [18]. 
